# Fenofibrate inhibits TGF‐β‐induced myofibroblast differentiation and activation in human lung fibroblasts *in vitro*

**DOI:** 10.1002/2211-5463.13247

**Published:** 2021-07-16

**Authors:** Ryota Kikuchi, Yuki Maeda, Takao Tsuji, Kazuhiro Yamaguchi, Shinji Abe, Hiroyuki Nakamura, Kazutetsu Aoshiba

**Affiliations:** ^1^ Department of Respiratory Medicine Tokyo Medical University Ibaraki Medical Center Inashiki Japan; ^2^ Department of Respiratory Medicine Tokyo Medical University Shinjuku‐ku Japan; ^3^ Department of Medicine Otsuki Municipal Hospital Japan

**Keywords:** fenofibrate, myofibroblasts, PPARα, pulmonary fibrosis, TGF‐β

## Abstract

Fenofibrate (FF), a peroxisome proliferator‐activated receptor‐alpha (PPAR‐α) agonist and a lipid‐lowering agent, can decrease experimental pulmonary fibrosis. However, the mechanisms underlying the antifibrotic effect of FF remain unknown. Hence, this study was conducted to evaluate the effects of FF on transforming growth factor‐beta (TGF‐β)‐induced myofibroblast differentiation and activation in lung fibroblasts. The results showed that FF inhibited alpha‐smooth muscle actin (α‐SMA) and connective tissue growth factor expression, collagen production, cell motility, SMAD3 phosphorylation and nuclear translocation, and metabolic reprogramming in TGF‐β‐exposed cells. The inhibitory effect of FF did not decrease with the addition of a PPAR‐α antagonist. Moreover, the inhibitory effect given by FF could not be reproduced with the addition of an alternative PPAR‐α agonist. FF inhibited mitochondrial respiration. However, rotenone, a complex I inhibitor, did not suppress TGF‐β‐induced myofibroblast differentiation. Furthermore, the TGF‐β‐induced nuclear reduction of protein phosphatase, Mg^2+^/Mn^2+^‐dependent 1A (PPM1A), a SMAD phosphatase, was inhibited by FF. These results showed that FF suppressed TGF‐β‐induced myofibroblast differentiation and activation independent of PPAR‐α activation and impaired mitochondrial respiration. In conclusion, this study provides information on the effects of FF on anti‐TGF‐β mechanisms.

## Graphical abstract

Fenofibrate, a peroxisome proliferator‐activated receptor‐α (PPAR‐α) agonist and a lipid‐lowering agent, inhibits transforming growth factor‐β (TGF‐β)‐induced myofibroblast differentiation via PPAR‐α‐independent mechanisms, which probably include the upregulation of protein phosphatase, Mg^2+^/Mn^2+^‐dependent 1A, a SMAD phosphatase, leading to export of nuclear SMAD3 to cytoplasm. This study provides evidence on the effects of fenofibrate on anti‐TGF‐β actions.

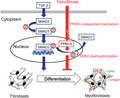

**Abbreviations**
CTGF: connective tissue growth factor
DMEM: Dulbecco's modified Eagle's medium
ECAR: extracellular acidification rate
FF: fenofibrate
OCR: oxygen concentration rate
PPAR‐α: peroxisome proliferator‐activated receptor‐alpha
PPM1A: protein phosphatase Mg^2+^/Mn^2+^‐dependent 1A
TGF‐β: transforming growth factor‐beta
α‐SMA: α‐smooth muscle actin

Myofibroblast differentiation and activation are the key pathogenic events involved in many pulmonary diseases, such as asthma, chronic obstructive pulmonary disease, and idiopathic pulmonary fibrosis [1, 2, 3, 4]. In these diseases, airway or parenchymal fibrosis is mechanistically mediated by common and/or disease‐specific pathways, among which transforming growth factor‐beta (TGF‐β) signaling plays a central role. TGF‐β induces the phosphorylation of SMAD proteins, stimulating signal transduction pathways that lead to myofibroblast differentiation and transcription factor activation and alpha‐smooth muscle actin (α‐SMA) and collagen production [5, 6, 7].

Fenofibrate (FF) is a widely used antihyperlipidemic agent exhibiting a lipid‐lowering effect by activating peroxisome proliferator‐activated receptor‐alpha (PPAR‐α) [8]. In addition, FF exerts pleiotropic actions on multiple pathways to reduce inflammation, oxidative stress, apoptosis, angiogenesis, and fibrosis [9, 10]. Notably, recent clinical studies on patients with diabetes have revealed that FF has protective effects on retinopathy and nephropathy [11, 12]. Moreover, several animal studies have shown that FF inhibits experimental fibrosis in different organs, including the retina, heart, liver, kidney, and lung [13, 14, 15, 16, 17, 18, 19, 20]. For example, the oral administration of FF in rats has been shown to attenuate the severity of bleomycin‐induced pulmonary fibrosis [20]. Since the overexpression of TGF‐β is critical in fibrotic disease, we hypothesized that FF inhibits the TGF‐β signaling pathway. The molecular mechanism of action of FF appears to be complex, which includes not only PPAR‐α‐dependent but also PPAR‐α‐independent mechanisms [21, 22, 23, 24, 25, 26, 27, 28, 29, 30, 31]. Thus, this study was conducted to assess the effects of FF on TGF‐β‐induced myofibroblast differentiation and activation *in vitro* and to determine whether the effects of FF depend on PPAR‐α.

## Methods

### Cell culture

Human fetal lung fibroblasts (IMR‐90; American Type Culture Collection, Manassas, VA, USA) were cultured and maintained in Dulbecco's modified Eagle's medium (DMEM; Gibco®; Thermo Fisher Scientific, Inc., Waltham, MA, USA) containing 25 mm glucose, 4 mm glutamine, and 10% FBS at 37 °C in a humidified incubator (CO_2_ incubator 900EX; Wakenyaku Co., Ltd., Tokyo, Japan) saturated with a gas mixture containing 5% CO_2_, ~ 20% O_2_, and 75% N_2_. Upon reaching confluence, the cells were cultured in serum‐free DMEM for 24 h, pretreated with FF (25 μm unless otherwise indicated; Sigma‐Aldrich Japan, Tokyo, Japan) or vehicle alone for 1 h, and then treated with 5 ng·mL^−1^ TGF‐β (PeproTech., Cranbury, NJ, USA) in the presence or absence of FF. In the relevant experiments, WY14643 (50 μm; Cayman Chemical, Ann Arbor, MI, USA), GW6471 (20 μm; Cayman Chemical), or rotenone (0.5 μm; Sigma‐Aldrich) was added to the culture medium. The concentrations of FF, TGF‐β, WY14643, and GW6471 used in this study were adopted from previous studies [32, 33, 34].

### Immunofluorescence staining

Cells in the Nunc® Lab‐Tek® eight‐well chamber slides (Thermo Fisher Scientific K.K., Yokohama, Japan) were fixed with 10% formalin and permeabilized with 0.3% Triton® X‐100 in PBS for 5 min. After blocking the nonspecific binding sites with 3% BSA, the slides were incubated with mouse monoclonal anti‐α‐SMA antibody (Novus Biologicals USA, Centennial, CO, USA) or rabbit monoclonal anti‐SMAD3 (Abcam Japan, Tokyo, Japan). Next, the primary antibody was allowed to react with a secondary anti‐mouse immunoglobulin G antibody conjugated with Alexa Fluor 488 (Invitrogen, Carlsbad, CA, USA). Then, the cell nuclei were counterstained with 4′,6‐diamidino‐2‐phenylindole. Fluorescence images were obtained using a microscope (Olympus IX71; Olympus Optical Co., Ltd., Tokyo, Japan) equipped with a digital camera.

### Western blotting

Cell samples were lysed in a radioimmunoprecipitation assay buffer (50 mm Tris hydrochloride, pH 7.4, 150 mm sodium chloride, 0.4 mm ethylenediaminetetraacetic acid, 0.5% Nonidet P‐40, and 0.1% SDS) containing a protease inhibitor cocktail (Sigma‐Aldrich Japan) and 1 mm sodium orthovanadate. According to the manufacturer's instructions, nuclear proteins were extracted using a nuclear extraction kit (Active Motif, Carlsbad, CA, USA). The samples were centrifuged at 10 000 ***g*** for 30 min, and the total protein concentrations in the supernatants were assessed using the DC protein assay kit (Bio‐Rad Laboratories, Hercules, CA, USA). Then, the samples (20 μg protein·lane^−1^) were then fractionated by SDS/PAGE, transferred to a polyvinylidene difluoride membrane (EMD Millipore Immobilon®‐P; Millipore Co., Billerica, MA, USA), and probed with the primary antibodies, such as rabbit polyclonal anti‐actin (Sigma‐Aldrich Japan), rabbit polyclonal anti‐lamin B1 (ProteinTech Group Inc., Rosemont, IL, USA), mouse monoclonal anti‐α‐SMA (Novus Biologicals USA), goat polyclonal anti‐connective tissue growth factor (CTGF; Santa Cruz Biotechnology, Inc., Santa Cruz, CA, the USA), rabbit monoclonal anti‐SMAD3 (Abcam Japan), rabbit monoclonal anti‐phospho‐SMAD3 (Ser423/425) (Abcam Japan), and rabbit polyclonal anti‐protein phosphatase, Mg^2+^/Mn^2+^‐dependent 1A (PPM1A; GeneTex, Inc., Irvine, CA, USA). The primary antibodies were detected using a horseradish peroxidase‐conjugated antibody, which was, in turn, visualized on enhanced chemiluminescence (SuperSignal West Pico; Pierce, Rockford, IL, USA). The signal intensities were quantified by densitometric scanning using imagej (version 1.49V; National Institutes of Health, Bethesda, MD, USA).

### Collagen assay

The collagen content in the culture medium was assessed using the Sircol® Soluble Collagen Assay Kit (Biocolor, Carrickfergus, UK).

### *In vitro* wound closure assay

The cells were cultured to reach confluence in 24‐well plates. After serum starvation for 24 h, they were scratched in a straight line using a sterile 200‐μL tip and were washed twice with PBS. Thereafter, the cells were incubated in serum‐free DMEM with or without TGF‐β (5 ng·mL^−1^) in the presence or absence of FF (25 μm). After 24 h, the cells were fixed with 10% formalin and were washed twice with PBS. Wound healing was assessed under a phase‐contrast microscope (Olympus IX71; Olympus Optical Co., Ltd.) equipped with a digital camera. The wound area was measured using image analysis software (Lumina Vision; Mitani Corporation, Fukui, Japan) on a Microsoft XP computer. Five measurements were taken from five fields of each well obtained from six wells in each experiment.

### Metabolic flux analysis

Cells were cultured in eight‐well XF assay plates and then treated with or without TGF‐β (5 ng·mL^−1^) in the presence or absence of FF (25 μm) or WY14643 (50 μm) for 48 h. The oxygen concentration rate (OCR) and extracellular acidification rate (ECAR) were measured using the Seahorse XFp Extracellular Flux Analyzer (Seahorse Bioscience, North Billerica, MA, USA).

### Assessment of glucose consumption

Glucose concentrations in the culture and original medium (not cultured with cells) were assessed using an amperometric blood glucose sensor (Nipro Care Fast C; Nipro Co., Osaka, Japan).

### Statistical analysis

Statistical analyses were performed using Microsoft Excel X with Statcel 3 (OMS; Tokyo, Japan) as an add‐in software. Data were expressed as means ± standard error of the mean. Equality of variance was examined using Bartlett's test. Data with equality of variance rejected were logarithmically transformed and retested. When equality of variance was recognized, statistical significance was analyzed using ANOVA. If significant results were obtained, the Tukey–Kramer test was used as a *post hoc* test for multiple comparisons. *P*‐values < 0.05 were used to denote statistically significant differences.

## Results

**Fig. 1:**
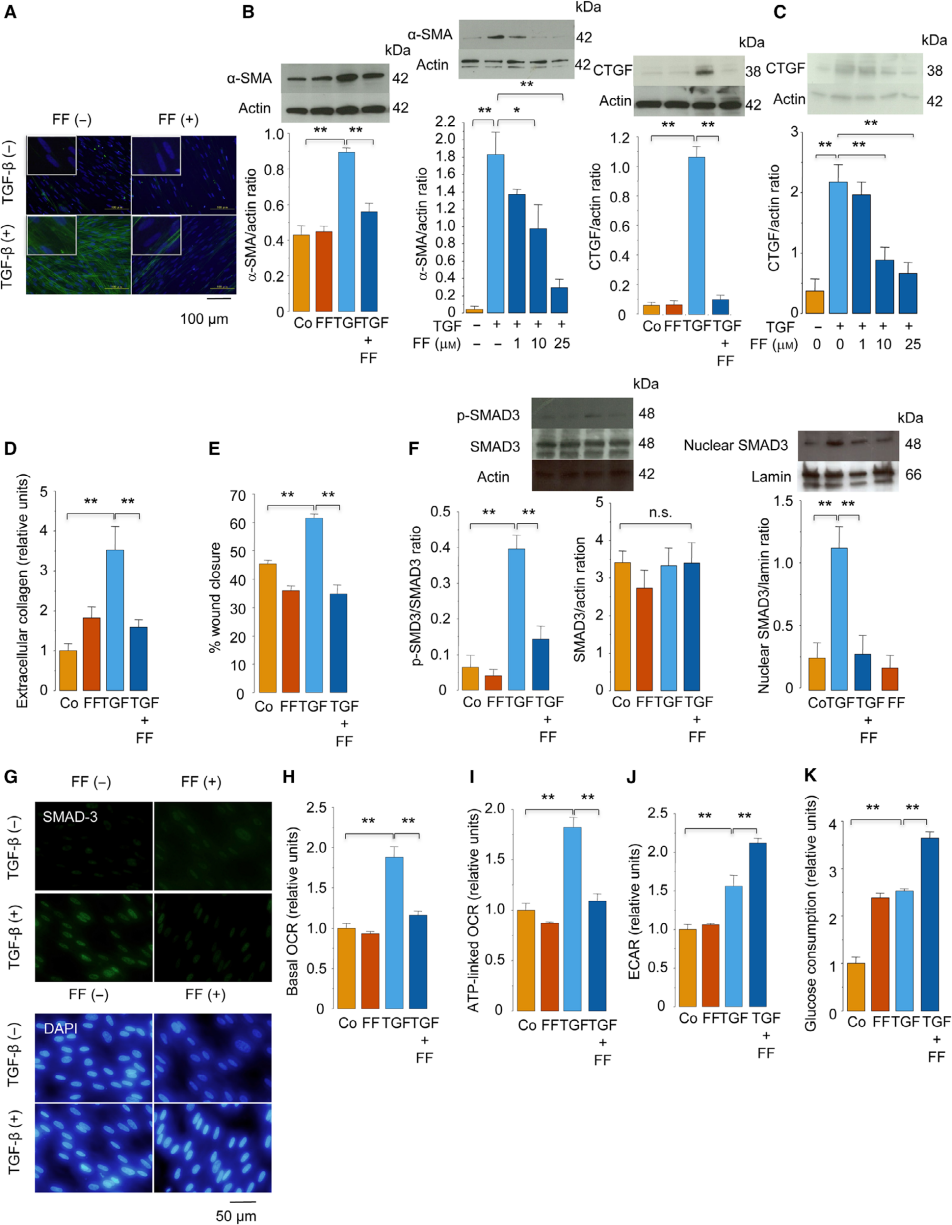
FF inhibits TGF‐β‐induced myofibroblast differentiation, collagen production, cell migration, SMAD3 phosphorylation, and metabolic reprogramming. IMR‐90 cells were pretreated with FF (25 μm unless otherwise indicated) or vehicle alone for 1 h and were treated with or without TGF‐β (5 ng·mL^−1^) in the presence or absence of FF for 1 h (F, G), 24 h (E), or 48 h (A–D, H–K). (A) Representative images of anti‐α‐SMA immunofluorescence (green, α‐SMA; blue, nuclear staining; *n* = 3). Insets are magnified images. Scale bar: 100 μm. (B, C) Detection of α‐SMA (B) and CTGF (C) using western blot analysis. The relative protein expression levels were evaluated using densitometry and were normalized to the expression level of β‐actin as the control (*n* = 4). (D) Extracellular collagen production assessed using the Sircol^®^ Soluble Collagen Assay (*n* = 4). (E) Wound closure assay. The migration of cells toward the wound was expressed as the percentage of wound closure (*n* = 4). (F) Western blot detection of phosphorylated SMAD3 and total SMAD3 (*n* = 4, left) and nuclear SMAD3 (*n* = 4, right). The relative protein expression levels were normalized to the expression level of β‐actin or lamin as the control. (G) Representative images of anti‐SMAD3 immunofluorescence (green) and nuclear staining with 4′,6‐diamidino‐2‐phenylindole (DAPI, blue; *n* = 3). Scale bar: 50 μm (H) Basal OCR (*n* = 3). (I) Adenosine triphosphate (ATP)‐linked OCR (*n* = 3). (J) ECAR (*n* = 3). (K) Glucose consumption rate (*n* = 4). Data were expressed as means ± standard error of the mean. *P < 0.05 and **P < 0.01 using the Tukey–Kramer test. Co, control; p‐SMAD3, phosphorylated SMAD3.

First, we examined whether FF modulates the effects of TGF‐β on IMR‐90 cells, a human lung fibroblast cell line. The treatment of IMR‐90 cells with TGF‐β (5 ng·mL^−1^) for 48 h enhanced the expression of α‐SMA, indicating myofibroblast differentiation (Fig. 1A,B). However, cotreatment with FF (1–25 μm) reduced the enhanced expression of α‐SMA in TGF‐β‐treated cells in a dose‐dependent manner. This finding showed that FF inhibits myofibroblast differentiation induced by TGF‐β. Moreover, treatment of IMR‐90 cells with TGF‐β increased the expression of CTGF (Fig. 1C), an important mediator of myofibroblast activation and extracellular matrix synthesis [35], and the production of collagen (Fig. 1D). Similar to the inhibition of α‐SMA expression by FF, cotreatment with FF also reduced the aforementioned enhanced CTGF expression and collagen production induced by exposure to TGF‐β (Fig. 1C,D). Since the activation of myofibroblasts is associated with increased cell motility [36], the effects of FF on cell migration were evaluated. The *in vitro* wound closure assay showed that the mobility of TGF‐β‐treated IMR‐90 cells was significantly decreased in the presence of FF (Fig. 1E). SMADs, including SMAD3, were found to play a critical role in TGF‐β signaling pathways leading to myofibroblast differentiation and extracellular matrix protein production [5]. Western blotting and immunofluorescence revealed that pretreatment with FF inhibited TGF‐β‐induced phosphorylation and nuclear translocation of the SMAD3 (Fig. 1F,G).

Myofibroblast differentiation is associated with metabolic reprogramming, which was proposed as a molecular target of TGF‐β action [37, 38]. According to previous reports [37, 38], treatment with TGF‐β increased both OCR, as evidenced by augmented basal respiration (Fig. 1H) and mitochondrial ATP production (Fig. 1I), and glycolysis, as indicated by increased ECAR (Fig. 1J) and glucose consumption (Fig. 1K). Cotreatment with FF decreased TGF‐β‐induced mitochondrial respiration (Fig. 1H,I). However, interestingly, it enhanced TGF‐β‐mediated stimulation of glycolysis (Fig. 1J,K). Taken together, these results showed that FF suppressed SMAD activation, myofibroblast differentiation, collagen production, cell mobility, and mitochondrial respiration in human lung fibroblasts treated with TGF‐β.

**Fig. 2:**
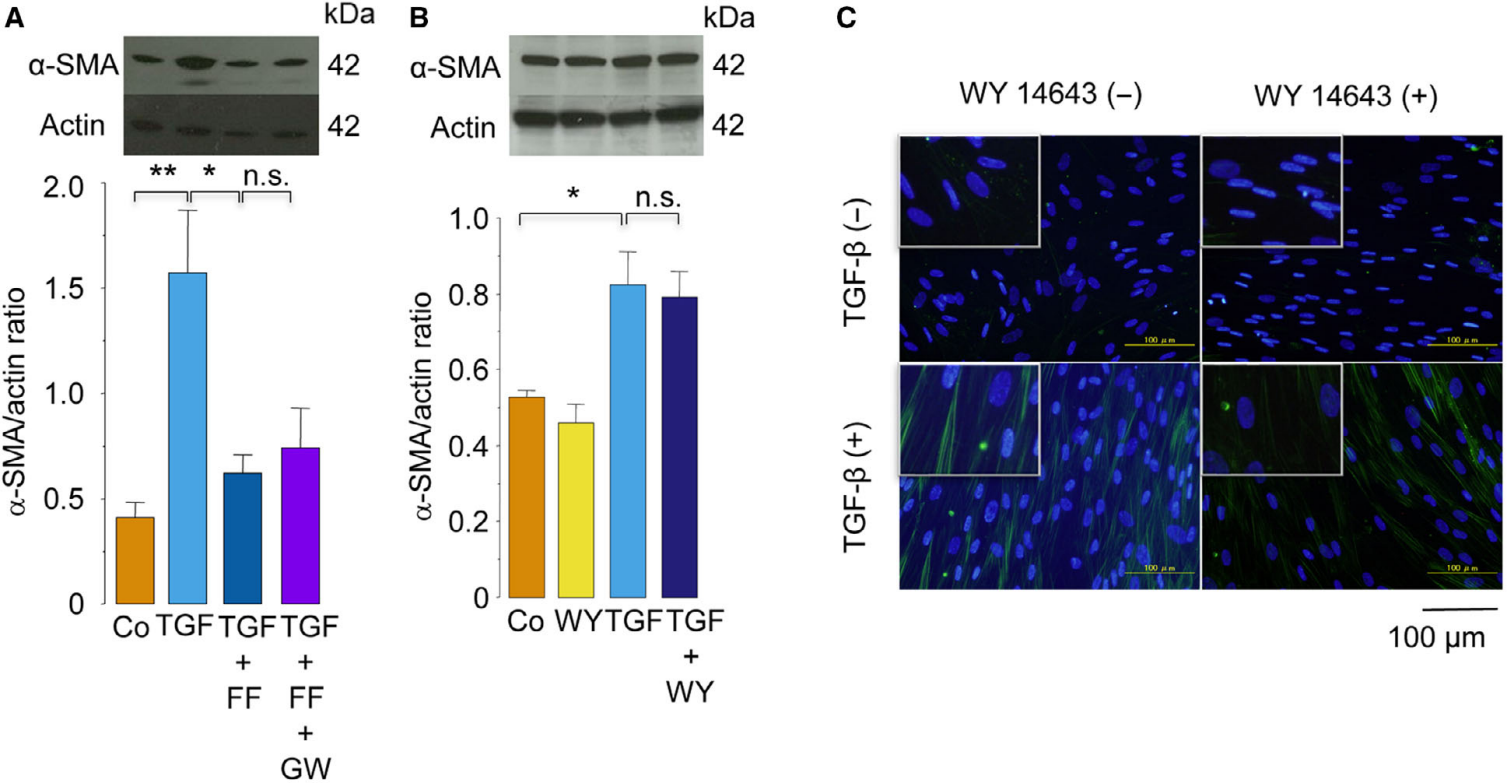
Inhibition of TGF‐β‐induced myofibroblast differentiation by FF was not attributed to the activation of PPAR‐α. (A) IMR‐90 cells were pretreated with vehicle alone, FF (25 μm), or FF plus GW6471 (20 μm) for 1 h and were treated with or without TGF‐β (5 ng·mL^−1^) for 48 h in the presence or absence of FF or GW6471. The expression of α‐SMA protein was evaluated using western blot analysis (*n* = 4). (B, C) IMR‐90 cells were pretreated with WY14643 (50 μm) or vehicle alone for 1 h and were then treated with or without TGF‐β (5 ng·mL^−1^) for 48 h in the presence or absence of WY14643. The expression of α‐SMA protein was evaluated using western blot analysis (B, *n* = 4) or immunofluorescence staining (C, *n* = 4). green, α‐SMA; blue, nuclear staining. Insets are magnified images. Scale bar: 100 μm. Data were expressed as means ± standard error of the mean. *P < 0.05 and **P < 0.01 using the Tukey–Kramer test. n.s., not significant. Co, control; GW, GW6471; WY, WY14643.

The lipid‐lowering effect of FF is mediated by the activation of PPAR‐α, a nuclear receptor to which FF binds as a ligand [39]. Thus, we next examined whether the inhibition of TGF‐β‐induced myofibroblast differentiation by FF was attributed to the activation of PPAR‐α. The results showed that treatment with GW6471 (20 μm), a PPAR‐α antagonist, did not change the inhibitory effect of FF on TGF‐β‐induced α‐SMA expression (Fig. 2A). Furthermore, treatment with WY14643 (50 μm), a PPAR‐α agonist, did not inhibit TGF‐β‐induced α‐SMA expression (Fig. 2B,C). These findings showed that the inhibition of TGF‐β‐induced myofibroblast differentiation by FF is mediated by a mechanism independent of PPAR‐α activation.

**Fig. 3:**
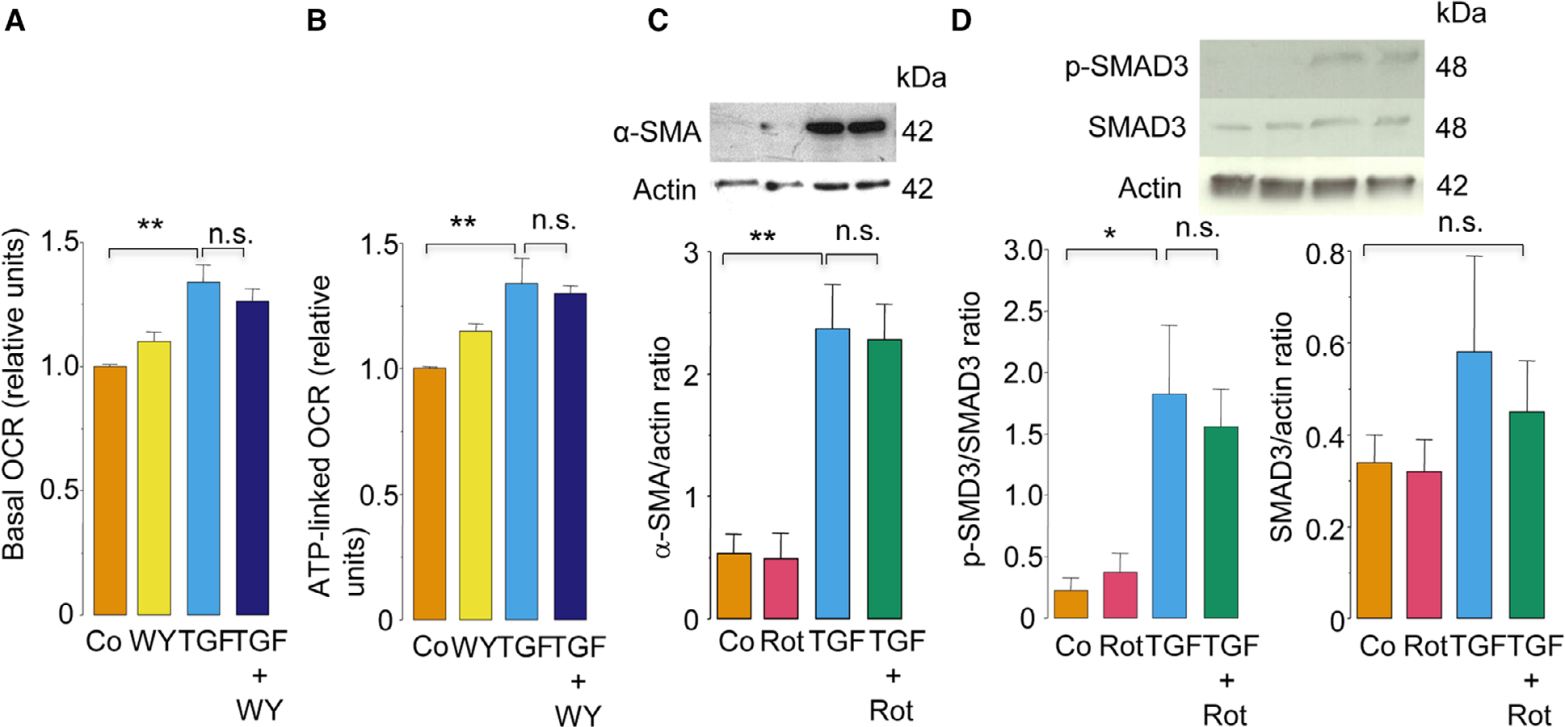
Inhibition of TGF‐β‐induced myofibroblast differentiation by FF was not attributed to the inhibition of mitochondrial respiration. (A, B) The effects of WY14643 on the basal OCR (A, *n* = 3) and ATP‐linked OCR (B, *n* = 3). IMR‐90 cells were pretreated with WY14643 (50 μm) or vehicle alone for 1 h and were then treated with or without TGF‐β (5 ng·mL^−1^) for 48 h in the presence or absence of WY14643. (C, D) Effects of rotenone on the expression of α‐SMA (C) and phosphorylation of SMAD3 (D). IMR‐90 cells were pretreated with rotenone (0.5 μm) or vehicle alone for 1 h and were then treated with or without TGF‐β (5 ng·mL^−1^) in the presence or absence of rotenone for 1 h (D) or 48 h (C). The α‐SMA expression (C) and SMAD3 phosphorylation (D) levels were evaluated using western blot analysis (*n* = 4). Data were expressed as means ± standard error of the mean. *P < 0.05 and **P < 0.01 using the Tukey–Kramer test. n.s., not significant. Co, control; WY, WY14643; Rot, rotenone; p‐SMAD3, phosphorylated SMAD3.

FF inhibits complex 1 of the mitochondrial electron transfer chain [40]. In this study, in contrast to FF, WY14643 did not suppress the high mitochondrial respiration induced by TGF‐β (Fig. 3A,B). Hence, it was unlikely that the inhibition of mitochondrial respiration by FF was caused by PPAR‐α activation. Then, we addressed the possibility that the PPAR‐α‐independent inhibition of mitochondrial complex I by FF is mechanistically involved in inhibiting TGF‐β‐induced myofibroblast differentiation. However, treatment with rotenone, a complex I inhibitor, did not inhibit either α‐SMA expression (Fig. 3C) or SMAD3 phosphorylation (Fig. 3D) in TGF‐β‐treated cells. This result indicated that the inhibition of TGF‐β‐induced myofibroblast differentiation by FF is not attributed to the inhibition of mitochondrial respiration.

**Fig. 4:**
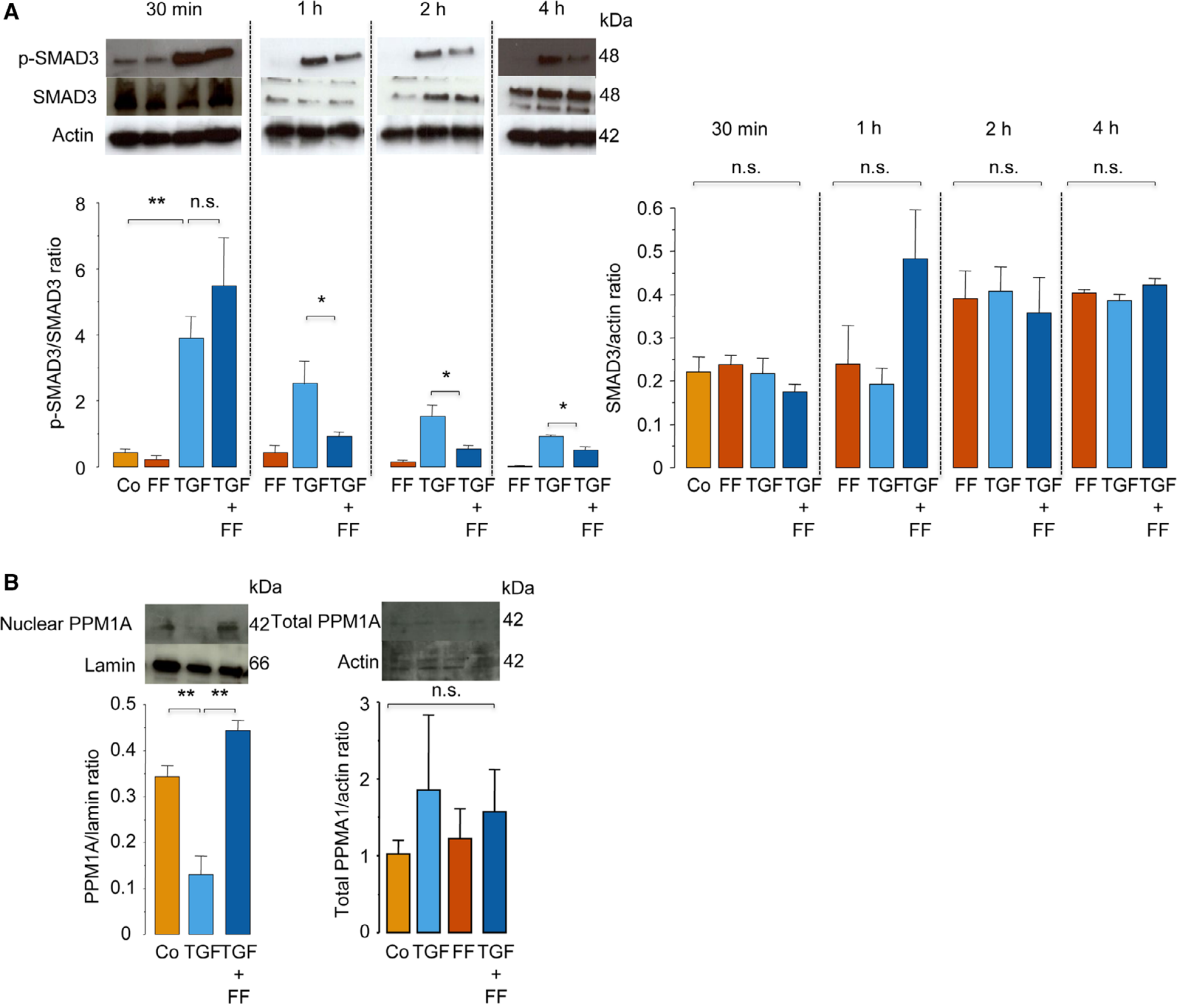
Effects of FF on SMAD3 phosphorylation (A) and the total and nuclear levels of PPM1A (B). IMR‐90 cells were pretreated with FF (25 μm) or vehicle alone for 1 h and were then treated with or without TGF‐β (5 ng·mL^−1^) in the presence or absence of FF for 30 min, 1, 2, and 4 h (A, *n* = 4) or 1 h (B, *n* = 4). Total cell lysates (A; B, right) or nuclear lysates (B, left) were analyzed using western blot analysis. Data were expressed as means ± standard error of the mean. *P < 0.05 and **P < 0.01 using the Tukey–Kramer test. n.s., not significant. Co, control; p‐SMAD3, phosphorylated SMAD3.

A time‐course analysis of FF inhibition of TGF‐β‐induced phosphorylation of SMAD3 revealed that FF reduced the cellular levels of phosphorylated SMAD3 after 1 h or thereafter but not as early as 30 min after treatment with TGF‐β. This result showed that FF might have reduced TGF‐β‐induced SMAD3 phosphorylation at a later time by dephosphorylating SMAD3 (Fig. 4A). Studies have shown that PPM1A dephosphorylates and promotes the nuclear export of SMAD3 to terminate TGF‐β signaling [41]. Lastly, we found that treatment with TGF‐β decreased the nuclear levels but not the total levels of PPM1A, which was partially recovered after cotreatment with FF (Fig. 4B). These findings suggest that FF partially inhibited TGF‐β‐induced reduction of nuclear PPM1A.

## Discussion

This study showed that treatment with FF inhibits TGF‐β‐induced myofibroblast differentiation and activation, as evidenced by the suppression of α‐SMA and CTGF expression, collagen production, cell mobility, SMAD3 phosphorylation and nuclear translocation, and mitochondrial respiration in human lung fibroblasts treated with TGF‐β. The lipid‐lowering effect of FF is reportedly mediated by activating PPAR‐α [39]. However, this study revealed that the inhibition of TGF‐β‐induced myofibroblast differentiation by FF is not caused by PPAR‐α activation because the PPAR‐α antagonist GW6471 did not suppress the inhibitory effect of FF on TGF‐β‐induced myofibroblast differentiation and treatment with the PPAR‐α agonist WY14643 did not inhibit TGF‐β‐induced myofibroblast differentiation.

Accumulating evidence indicates that PPAR‐α‐independent mechanisms are involved in the pleiotropic effects of FF on various physiological and pathological processes [21, 22, 23, 24, 25, 26, 27, 28, 29, 30, 31, 42]. For example, FF has been shown to inhibit tumor cell proliferation [21, 23, 28] and diapedesis [25], angiogenesis [24], endothelin‐1 expression by endothelial cells [26], renal organ cation transporter [27], and insulin secretion [30], which are reportedly mediated independently of PPAR‐α. Concerning the mechanism of the independent PPAR‐α action of FF, many molecular targets have been proposed, such as nuclear factor‐kappa B [23], Akt [21, 28], Stat3 [22], cytochrome P450 2C [24], glycogen synthase kinase‐3 [26], growth differentiation factor‐15 [29], and ATP‐sensitive potassium channel [30].

This study showed that FF inhibits mitochondrial respiration via a PPAR‐α‐independent mechanism. However, the inhibition of TGF‐β‐induced myofibroblast differentiation by FF was not attributed to the inhibition of mitochondrial respiration because rotenone did not suppress TGF‐β‐induced myofibroblast differentiation. This study also showed that FF partially inhibits TGF‐β‐induced reduction of nuclear PPM1A, a SMAD phosphatase that terminates TGF‐β signaling [41]. Thus, the FF‐mediated regulation of PPM1A may mechanistically be accounted at least in part for the FF‐mediated inhibition of TGF‐β‐induced myofibroblast differentiation. Interestingly, Paw *et al*. [32] have recently shown that FF inhibits TGF‐β‐induced myofibroblast differentiation by disturbing the organization of actin cytoskeleton architecture. They have found that FF inhibits TGF‐β‐stimulated incorporation of α‐SMA into stress fibers by reducing connexin 43, a protein that constitutes gap junctional channels. However, in contrast to this study, they have found that FF does not suppress TGF‐β‐induced α‐SMA expression despite the inhibition of SMAD2/3 signaling. The discrepancy between their study and this study may be attributed to different sources of fibroblasts: We used fibroblasts derived from normal fetal lungs, whereas Paw *et al*. used bronchial fibroblasts derived from patients with asthma. Although the results of this study indicated PPAR‐α‐independent mechanisms by which FF suppresses TGF‐β‐induced myofibroblast differentiation, we still considered the possibility that PPAR‐α activation has some antifibrotic effects because PPAR‐α‐knockout mice treated with bleomycin had more severe inflammation and injury than wild‐type mice [43].

Recent animal studies have shown that treatment with FF decreases experimental fibrosis in the retina, lungs, liver, heart, and kidneys [13, 14, 15, 16, 17, 18, 19, 20]. FF is a relatively safe and inexpensive agent widely used in clinical practice for treating hyperlipidemia. This study provided a better understanding of the antifibrotic effect of FF, which can be used as a basis for its clinical application for treating fibrotic disorders.

## Author contributions

RK and KA conceived and designed the project. RK, YM, and KA acquired, analyzed, and interpreted the data. RK and KA wrote the original draft. TT. KY, SA, and HN revised critically and edited the draft.

## Conflict of interest

The authors declare no conflict of interest.

## Data Availability

The analyzed datasets generated during this study are available from the corresponding author on reasonable request.
